# mSphere of Influence: Organoids and Single-Cell Sequencing, a Powerful Combination To Uncover Epithelial and Immune Cell Interactions in the Human Gut Environment

**DOI:** 10.1128/mSphere.00722-20

**Published:** 2020-07-29

**Authors:** Jose M. Lemme-Dumit

**Affiliations:** a Department of Pediatrics, Center for Vaccine Development and Global Health, University of Maryland School of Medicine, Baltimore, Maryland, USA

**Keywords:** enteroid, immune cells, mucosal immunology, single-cell sequencing

## Abstract

Jose Lemme-Dumit works in the field of mucosal immunology and vaccines. In this mSphere of Influence article, he reflects on how two papers, “Long-term expansion of epithelial organoids from human colon, adenoma, adenocarcinoma, and Barrett’s epithelium” by Sato et al. (T. Sato, D. E. Stange, M. Ferrante, R. G. J. Vries, et al., Gastroenterology 141:1762–1772, 2011, https://doi.org/10.1053/j.gastro.2011.07.050) and “T helper cell cytokines modulate intestinal stem cell renewal and differentiation” by Biton et al. (M. Biton, A. L. Haber, N. Rogel, G. Burgin, et al., Cell 175:1307–1320.E22, 2018, https://doi.org/10.1016/j.cell.2018.10.008), have influenced his research by describing the development of intestinal organoid cultures and implementation of high-throughput sequencing analysis. The combination of these forefront technologies has expanded opportunities for mechanistic interrogation of host immunity to enteric pathogens.

## Abstract (precis)

Jose Lemme-Dumit works in the field of mucosal immunology and vaccines. In this mSphere of Influence article, he reflects on how two papers, “Long-term expansion of epithelial organoids from human colon, adenoma, adenocarcinoma, and Barrett’s epithelium” by Sato et al. (T. Sato, D. E. Stange, M. Ferrante, R. G. J. Vries, et al., Gastroenterology 141:1762–1772, 2011, https://doi.org/10.1053/j.gastro.2011.07.050) and “T helper cell cytokines modulate intestinal stem cell renewal and differentiation” by Biton et al.

## COMMENTARY

Homeostasis and host defenses in the human gut are finely regulated. The intestinal epithelium provides a physiological and immunological barrier that protects the host from pathogens and harmful agents while maintaining a peaceful coexistence with nutrients and the gut microbiota. A variety of epithelial cell culture models have been used to study biological processes in the human gut. The majority of these *in vitro* models employ cancer-derived cell lines. Although their use is practical, the genetic modifications and aberrant behavior of immortalized cells render results unreliable and nongeneralizable. Other models involve *ex vivo* primary cultures. The limited viability of human primary cells restricts and limits their use. A major advancement in tissue culture technology was achieved by the identification of the “stemness” marker Lgr5 (leucine-rich-repeat-containing G-protein-coupled receptor 5) at the intestinal crypts (1). This breakthrough transformed tissue culture technologies by enabling the generation of three-dimensional (3D) self-organizing tissue structures that recapitulate critical features of the human intestinal epithelium *in vivo*. Self-renewal of the intestinal epithelium requires distinct proliferative signals on crypt intestinal stem cells. In the research article “Long-term expansion of epithelial organoids from human colon, adenoma, adenocarcinoma, and Barrett’s epithelium” (2), Clevers’ group identified the essential factors necessary for *ex vivo* expansion and differentiation of Lgr5-positive (Lgr5^+^) human intestinal stem cells and for establishment of long-term organoid cultures. These factors included ligands for Wnt signaling (i.e., Wnt3A and R-spondin 1) and epidermal growth factor, both of which are necessary for intestinal stem cell proliferation, as well as Noggin (a bone morphogenetic protein inhibitor), an inducer of cell expansion. The authors found that gastrin and nicotinamide improved culture efficiency, with nicotinamide being essential for prolonging culture viability for up to 1 month. Further, inhibition of MAPK (p38) and transforming growth factor β (TGF-β) type 1 (Alk4/5/7) signals increased intestinal stem cell stability and expansion, extending the duration of cultures by at least 6 months. Besides identifying essential factors for intestinal stem cell expansion, Sato and colleagues showed that organoids retain essential aspects of the tissue from which they were derived, including aspects such as architecture, cell type composition, self-renewal dynamics, and normal karyotype. Organoids can be biobanked, which adds practicality. The technology described in that seminal paper has been broadly applied to produce organoids from other polarized epithelial organs such as stomach, lung, bile duct and pancreas, and prostate and mammary gland. Since the original publication of that paper, conditions appropriate for use in establishing 2D organoids (also known as enteroids) have been described. Advantages of working with polarized epithelial cells in a steady monolayer include ease of handling and direct access to apical and basolateral compartments for treatment and analysis.

The discoveries by Clevers’ group detailed in their report and follow-up studies have inspired and catalyzed not only my research but an entire field of study. Avoiding the drawbacks represented by cancer cell lines and the difficulty of working with animals and their host restriction, their approach opened a new avenue to the investigation of gut physiology and, in my case, of host-pathogen interactions and mucosal immunity. The organoid system as originally described still lacks the major cells and components that support and interact with the intestinal epithelium and that have important functional roles: innervation, fibroblasts and extracellular matrix components, and immune cells. My work has focused on the development and characterization of human enteroid-immune cell cocultures and the interrogation of immune mechanisms of host defense against enteric pathogens.

The topic of the complexity of the molecular and cellular processes involved in mucosal immunity to enteric organisms brings me to my second selected paper, which illustrates the unprecedented opportunity offered by recent technological advances to fill important knowledge gaps. Cutting-edge tools, including cell imaging, gene editing, and molecular analyses such as single-cell RNA sequencing (scRNA-seq), are now available for deep mechanistic interrogation of coordinated multicellular host responses. In the research article “T helper cell cytokines modulate intestinal stem cell renewal and differentiation” (3), the authors employed scRNA-seq analysis to reveal a mechanistic relationship between T helper (Th) cells and intestinal stem cells that orchestrates tissue-wide responses to external signals. Single-cell sequencing analysis generates a high-resolution profile of individual cell function within the tissue microenvironment. Taking advantage of this approach and employing organoids along with other systems, Biton and colleagues deciphered a nonconventional antigen-presenting role of intestinal stem cells and the influence of Th cells on epithelial cell renewal. Those investigators identified three Lgr5^+^ intestinal stem cell subsets with different proliferative capacities capable of interacting with and activating naive Th cells via major histocompatibility complex class II (MHCII) antigen presentation. Treg and interleukin-10 (IL-10) were found to promote organoid expansion, while Th1, Th2, and Th17 cells and derived cytokines (i.e., IL-13 or IL-17) depleted intestinal stem cells and induced organoid differentiation. In addition, luminal signals (bacteria or parasite) influenced the intestinal stem cell-Th axis and shaped epithelial cell composition, increasing levels of MHCII-expressing stem cells. While the work was conducted using murine organoid and intestinal mouse crypts, the report highlights an important form of epithelial and immune cell cross talk as a regulated response for maintenance of epithelial self-renewal or for promotion of its differentiation. These results challenge the prevailing paradigm of adaptive immune cell function by showing that they not only contribute to antimicrobial surveillance but also support intestinal tissue growth and differentiation. The role of Th cells activated by intestinal stem cells in host immunity and tolerance is wide open for investigation. Single-cell analysis is the “gold standard” for discerning cell responses and interaction. The approach of Biton and colleagues stimulated ideas that I am applying in my own research to decipher interactions between the human intestinal epithelium and immune cells in the steady state, in response to infection, and during the phase of recovery, facilitating tissue repair.

The two elegant publications discussed here illustrate the impact of cutting-edge technologies and their potential to advance translational research. Organoids and single-cell analyses could be powerful tools for personalized medicine; they would allow evaluation of drugs or therapies, understanding of genetic risks or disease susceptibility, and even correction of inherited defects.
